# Dr. Eugene Adelbert Chapman

**Published:** 1917-03

**Authors:** 


**Dr. Eugene Adelbert Chapman,** Buffalo 1862, died at his home in Stafford Springs, Conn., of cerebral haemorrhage, Jan. 29, aged 77. He was a veteran of the Civil War and subsequently Captain and Asst. Surgeon in the U. S. Army, serving as quarantine officer at Brazos, Tex., in 1865. He resided for many years at Henderson, Jefferson Co., N. Y., being postmaster in 1872-3, coroner in 1870 and 1886, county clerk 1900-1906.

